# Age-dependent loss of *Crls1* causes myopathy and skeletal muscle regeneration failure

**DOI:** 10.1038/s12276-024-01199-x

**Published:** 2024-04-01

**Authors:** Youngbum Yoo, MyeongHoon Yeon, Won-Kyung Kim, Hyeon-Bin Shin, Seung-Min Lee, Mee-Sup Yoon, Hyunju Ro, Young-Kyo Seo

**Affiliations:** 1 Aging Convergence Research Center, Korea Research Institute of Bioscience and Biotechnology (KRIBB), Daejeon, 34141 Republic of Korea; 2 Department of Biological Sciences, College of Bioscience and Biotechnology, Chungnam National University, Daejeon, 34134 Republic of Korea; 3 Biomolecular Science, KRIBB School of Bioscience, Korea University of Science and Technology (UST), Daejeon, 34141 Korea; 4 Department of Molecular Medicine, College of Medicine, Gachon University College of Medicine, Incheon, 21999 Republic of Korea; 5 Department of Health Sciences and Technology, GAIHST, Gachon University, Incheon, 21999 Republic of Korea; 6 School of Medicine, Sungkyunkwan University, Suwon, 16419 Republic of Korea

**Keywords:** Cell biology, Gene expression analysis, Predictive markers

## Abstract

Skeletal muscle aging results in the gradual suppression of myogenesis, leading to muscle mass loss. However, the specific role of cardiolipin in myogenesis has not been determined. This study investigated the crucial role of mitochondrial cardiolipin and cardiolipin synthase 1 (Crls1) in age-related muscle deterioration and myogenesis. Our findings demonstrated that cardiolipin and Crls1 are downregulated in aged skeletal muscle. Moreover, the knockdown of *Crls1* in myoblasts reduced mitochondrial mass, activity, and OXPHOS complex IV expression and disrupted the structure of the mitochondrial cristae. AAV9-shCrls1-mediated downregulation of *Crls1* impaired muscle regeneration in a mouse model of cardiotoxin (CTX)-induced muscle damage, whereas AAV9-mCrls1-mediated *Crls1* overexpression improved regeneration. Overall, our results highlight that the age-dependent decrease in CRLS1 expression contributes to muscle loss by diminishing mitochondrial quality in skeletal muscle myoblasts. Hence, modulating CRLS1 expression is a promising therapeutic strategy for mitigating muscle deterioration associated with aging, suggesting potential avenues for developing interventions to improve overall muscle health and quality of life in elderly individuals.

## Mitochondrial Cardiolipin: The Unsung Hero in Age-Related Muscle Deterioration

Aging often results in a decrease in muscle mass and function, a condition called sarcopenia. This research examines the role of a protein found in mitochondria (the energy factories of cells), cardiolipin synthase 1 (CRLS1), in muscle health and aging. The scientists discovered that levels of CRLS1 and cardiolipin, a fat it produces, decrease in the muscles of older mice. When CRLS1 levels were artificially lowered in young mice, their muscle mass and strength reduced. On the other hand, increasing CRLS1 levels in older mice improved muscle mass and strength. The research also found that CRLS1 is essential for muscle cell development and mitochondrial function. These results suggest that CRLS1 could be a potential treatment target for sarcopenia. More research is needed to further understand the metabolic changes caused by CRLS1 regulation.

This summary was initially drafted using artificial intelligence, then revised and fact-checked by the author.

## Introduction

Skeletal muscle is a highly organized tissue that is responsible for movement and maintaining overall physical strength. This tissue comprises approximately 40% of the human body weight and contains 50–75% of all body proteins^1^. With aging, skeletal muscle mass and function tend to decline, leading to sarcopenia—an involuntary and age-associated loss of skeletal muscle. Sarcopenia is a disease that can rapidly develop in individuals as young as 50 years and, most commonly, older than 65 years^2–4^. Sarcopenia has various etiologies, including failed homeostatic control of protein synthesis and degradation and decreased intracellular mitochondrial function, leading to muscle loss^5–9^. Altered skeletal muscle mass and strength are partially attributable to an imbalance between protein synthesis and breakdown and other cellular components, such as mitochondrial dysfunction^10,11^. Among these factors, mitochondrial proteins have emerged as critical regulators of skeletal muscle homeostasis and regeneration. One such protein, cardiolipin synthase 1 (CRLS1), is an enzyme responsible for synthesizing cardiolipin (CL), a unique phospholipid predominantly found in the inner mitochondrial membrane. Considering that mitochondrial crista capacity impacts oxidative phosphorylation (OXPHOS)^12^, disruptions in crista morphology or altered CL levels can impair OXPHOS efficiency, leading to mitochondrial dysfunction^13^.

**Fig. 1:**
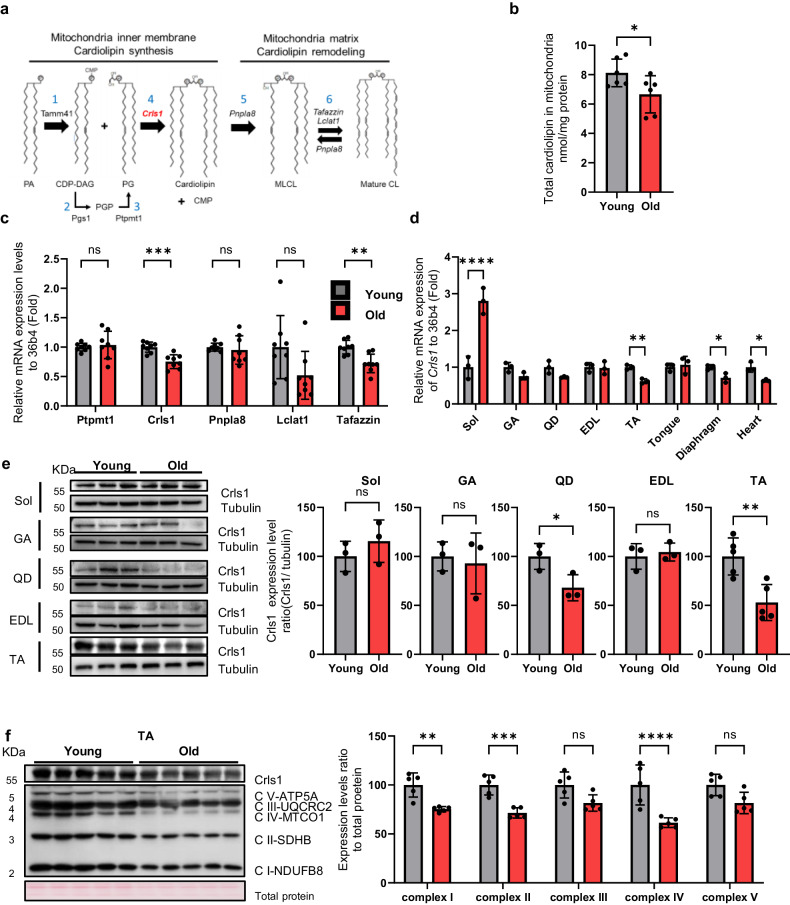
Diminished cardiolipin levels result in age-related muscle loss. **a** Schematic showing cardiolipin biosynthesis and remodeling in the mitochondrial matrix. **b** Total cardiolipin levels in the mitochondria of TA muscle from young and aged mice (*n* = 6 per group). **c** qPCR analyses of the expression of cardiolipin synthesis markers (Ptpmt1, Crls1) and remodeling genes (Pnpla8, Lclat1 and Tafazzin) in young and old TA muscle (*n* = 8 per group). **d** qPCR analysis of Crls1 gene expression in young and old mouse tissue. Sol: soleus, GA: gastrocnemius, QD: quadriceps, EDL: extensor digitorum longus, TA: tibialis anterior, heart, tongue, diaphragm. (*n* = 3 per group). **e** Immunoblots showing total Crls1 and mitochondrial complex protein levels in young and old hindlimb skeletal muscle. Mitochondrial complex and Crls1 protein expression levels relative to tubulin protein in young and old hindlimb skeletal muscle. (*n* = 3 per group). **f** Immunoblots of total Crls1 and mitochondrial complex protein from young and old TA skeletal muscle. Mitochondrial complex and Crls1 protein expression levels relative to total protein in young and old TA skeletal muscle. (*n* = 5 per group). The data are presented as the mean ± standard error of the mean (SEM) of *n* ≥ 3 independent experiments. *P* values were calculated using an unpaired Student’s t test. *P < 0.05; **P < 0.01; ***P < 0.001; n.s.: nonsignificant. Abbreviations: C57Bl/6j young (4 to 5 mo.) and aged (>22 mo.) mice were used. TA tibialis anterior, EDL extensor digitorum longus, GA gastrocnemius, QD quadriceps.

Mitochondrial energy production depends strongly on the composition of membrane phospholipids, specifically CL^14,15^. The inner membrane mitochondria (IMM) is the location of the CL biosynthesis and remodeling pathway. Phosphatidic acid (PA) is imported from the ER and transported across the inner membrane space via the protein complex PRELID–TRIAP1. After activation of PA by the CDP–DAG synthase TAMM41, phosphatidylglycerol phosphate synthase (PGS1) catalyzes the committed step by converting cytidine diphosphate diacylglycerol (CDP–DAG) to phosphatidylglycerol phosphate (PGP). Phosphatidylglycerol (PG) is formed by the phosphatase protein-tyrosine phosphatase mitochondrial 1 (PTPMT1). A second molecule, CDP–DAG, is used by cardiolipin synthase (CLS1) to form cardiolipin (CL)^16^, as shown in Fig. 1a. CL interacts with numerous proteins, enzymes and metabolite transporters within the IMM^17,18^. CL is a major lipid component that enhances mitochondrial oxidative capacity and is required for optimal complex I–IV activity^19–22^. Although the specific role of CL in skeletal muscle has not been determined, CL promotes the development of cristae and mitochondrial quality^23^. Thus, we hypothesized that altered CL levels in muscle cells causes functional impairment of the mitochondrial OXPHOS complex. Specifically, age-dependent loss of CRLS1 contributes to impaired muscle regeneration and the development of myopathy.

In the present study, we investigated the relationship between CL alterations and the age-dependent development of myopathy. Moreover, we explored the therapeutic potential of CL metabolism for mitigating skeletal muscle dysfunction. To test our hypothesis, we modulated CRLS1 expression in myoblast cell lines and a murine model. Our findings provide novel insights into the impact of age-dependent CRLS1 loss on muscle health and suggest potential therapeutic targets for skeletal muscle myopathy.

## Materials and Methods

### Cell culture

The mouse myoblast (C2C12) cell line was purchased from the American Type Culture Collection (ATCC, CRL-1772) and grown in Dulbecco’s modified Eagle medium (DMEM; Sigma) supplemented with 10% fetal bovine serum (FBS; Gibco) and 100 IU/mL penicillin‒streptomycin (P/S; Gibco). The cells were cultured in 5% CO_2_ at 37 °C. C2C12 cells were differentiated into myotubes using DMEM containing 2% horse serum (Gibco) for 24–120 h and transfected (Origene, TT320001) with siRNA duplexes targeting the *Crls1* gene or scrambled control siRNA (5.5–11 nM/6 well; Origene). After transfection, the myoblasts were differentiated for 120 h.

### Animal care

Young (3 to 5-month-old) and old (22-month-old) C57BL/6 J mice were purchased from the Laboratory Animal Resource Center (Korea Research Institute of Bioscience and Biotechnology [KRIBB]). The animal experiments were approved by KRIBB-IACUC. All mice were provided a standard laboratory diet (3.1 kcal/g) purchased from Damul Science (Daejeon, Korea). Complete randomization was applied for group assignment and experimental selection.

### Cardiotoxin-induced TA muscle injury mouse model

Cardiotoxin (CTX) was purchased from Sigma, dissolved in phosphate-buffered saline (PBS; 200 mM; Welgene), and injected into the TA muscle at 20 mM/50 μL/muscle using an insulin syringe (BD Ultra-Fine, 30 G, 0.5 mL). Fourteen days postinjury, the TA muscles were meticulously dissected using surgical instruments and either homogenized or carefully inserted into molds for further analysis.

### Viral infection of TA muscles

AAV9 was purchased from Vector Builder, Inc. (USA). We used AAV9-U6-promoter-shRNA-mCrls1-targeted virus (AAV9-shCrls1) and AAV9-MCK7-promoter-mCrls1-p2a-GFP (AAV9-mCrls1-GFP) virus. After dilution in PBS, AAV9-shCrls1 virus (1 × 10^11^/50 μL/muscle, i.m.) or AAV9-mCrls1-GFP virus (5 × 10^10^/50 μL/muscle, i.m.) was injected into the TA muscle.

### Plasmids and viruses for in vitro analyses

The pLKO.1 vector, mouse *Crls1*-targeting shRNA, and mCrls1-mCherry expression insert were purchased from Vector Builder, Inc. The target sequence was 5′-GCATCAGCATACAGTTATTAT-3′. HEK293T cells were transfected with mouse *Crls1*-targeted shRNA and the *Crls1*-mCherry expression plasmid using Lipofectamine 3000 (Thermo Fisher) for 16 h. The transfected cells were cultured for 24 h in DMEM supplemented with 10% FBS and 1% P/S to produce viruses, which were subsequently passed through a 0.45-μm polyethersulfone (PES) syringe filter. A filtered lentivirus (IPTG-inducible shRNA vector) was used to infect the C2C12 cell line (passage 4) in the presence of polybrene (1 μg/mL). Additionally, the cells were selected using blasticidin S HCl (5 μg/mL) for one week. Resistant cells were cultured in DMEM supplemented with IPTG (1 mM).

### Immunoblotting

Protein was extracted from whole-cell lysates and tissues with RIPA lysis buffer (50 mM Tris/HCl (pH 8.0), 150 mM NaCl, 1% NP-40, and 0.1% SDS) containing a protease and phosphatase inhibitor cocktail (Roche). After homogenization using FastPrep24TM (MP Biomedicals™) or sonication (Fangxu), the cell and tissue lysates were incubated on ice for 30 min and centrifuged at 14,000 ×g for 20 min at 4 °C. The supernatant was collected, and protein concentration was determined using the Pierce™ Rapid Gold BCA Protein Assay Kit (Thermo Fisher) before the addition of 5× SDS sample buffer containing 2-mercaptoethanol (Bio-Rad). Total proteins(10–20 mg) were separated on an SDS‒polyacrylamide gel according to the standard procedure at 25 mA per gel and blotted onto a 0.2 μm PVDF membrane (Bio-Rad) via the Mini Trans-blot module (Bio-Rad) at a constant voltage (250 mA) for 2 h. After blocking with 3% bovine serum albumin in Tris-buffered saline with 0.05% Tween 20 (TBS-T) for 1 h, the membrane was incubated overnight in 5% BSA/TBS-T with primary antibodies (Table S1). The membranes were then incubated with secondary-specific antibodies (Table S1). The membranes were scanned using a Luminograph II (ATTOKorea).

### Live-cell imaging

C2C12 cells were stained with MitoTracker™ Green FM (500 nM; Thermo Fisher) for 30 min and incubated in HEPES at 37 °C in a 5% CO_2_ incubator. The cells were then washed thrice with HEPES. Nuclei were stained with Hoechst 33342 dye (1:100,000; Invitrogen) for 15 min in HEPES at 37 °C and 5% CO_2_. Fluorescence was detected on an EVOS M5000 (Thermo Fisher).

### Immunocytochemistry

Differentiated C2C12 cells were washed with PBS twice and fixed in 4% paraformaldehyde (PFA) for 10 min. Fixed cells were washed in PBS and permeabilized with 0.3% PBST (Triton X-100) for 10 min. Cells were blocked with blocking buffer (PBS with 3% BSA) for 30 min before incubating with primary antibodies overnight at 4 °C with blocking buffer. The cells were then washed with 0.1% PBST (Tween 20) three times, incubated with the appropriate secondary antibodies for 1 h at room temperature (RT), and washed with 0.1% PBST (Tween 20) four times. The nuclei were stained with DAPI solution (VECTASHIELD® Antifade Mounting Medium with DAPI), and fluorescence was detected with an M5000 (Thermo Fisher). The myosin heavy chain-positive (MyhC + ) signal intensity was quantified using ImageJ software.

### Immunohistochemistry (IHC)

Frozen muscle tissues were embedded in optimal cutting temperature compound and sectioned (10 μm) using a cryostat (Leica). The tissue sections were fixed in 4% PFA for 10 min, washed in PBS for 10 min, permeabilized with 0.3% PBST (Triton X-100) for 10 min, blocked with blocking buffer (PBS with 10% goat serum) for 30 min, and incubated with primary antibodies specific for laminin for 2 h at RT. The tissues were washed three times with PBS before incubating with secondary antibodies for 1 h at RT and washed with PBST (Tween 20, 0.1%). The tissue slides were mounted with DAPI (VECTASHIELD® Antifade Mounting Medium with DAPI), and fluorescence was detected with an EVOS M5000 (Thermo Fisher).

### qRT‒PCR

Total RNA was extracted from cells and tissues using TRIzol reagent (Invitrogen) according to the manufacturer’s instructions. Reverse transcription was performed using a cDNA synthesis kit (Enzynomics). Quantitative real-time PCR was performed for target and endogenous genes (Table S2) using StepOne Plus (Applied Biosystems) with PowerUp SYBR Green Master Mix (Thermo Fisher). *TA, GA* muscle and *C2C12* expression levels were normalized to those of *36b4*. The fold change was calculated using the 2^-ΔΔCt^ method.

### Flow cytometric analysis of the mitochondrial membrane potential and mass

Scramble C2C12 cells and *Crls1*-knockdown C2C12 cells were harvested with a cell scraper and washed with PBS. Resuspended C2C12 cells were immediately stained with MitoTracker Green (500 nM) for 30 min at RT protected from light. The stained cells were washed with PBS containing 10% FBS and immediately analyzed via flow cytometry (BD Biosciences). The data analysis was performed using FlowJo.

### Total cardiolipin assay

Myoblasts or myotubes were washed with PBS. After resuspension in cardiolipin assay buffer (1 mL per 100 mm dish), the cells were transferred to a new 1.5 mL E-tube and homogenized with a sonicator. After centrifugation at 10,000× *g* for 10 min at 4 °C, the supernatant was transferred to a new 1.5 mL E-tube. The extracted cardiolipin concentration was measured using a cardiolipin assay kit (Abcam) according to the manufacturer’s instructions; the concentration was calculated using the following formula: detection value (nmol)/sample volume (μL) × sample dilution factor of the standard curve.

### Mitochondrial total cardiolipin assay

The TA muscle was dissected from the mouse hindlimb and homogenized using the Minute™ Mitochondria Isolation Kit for Muscle Tissues (invent BIOTECHNOLOGIES). Isolated mitochondria were homogenized with a sonicator in cardiolipin assay buffer. Next, 10–20 μL of homogenized mitochondria was added to the cardiolipin assay plate, and the concentration of cardiolipin was measured with a cardiolipin assay kit (Abcam) according to the manufacturer’s instructions. The measured cardiolipin concentration was calculated using the following formula: detection value (nmol)/sample volume (μL) × sample dilution factor of the standard curve.

### Cardiolipin staining of myoblasts

The cells were washed with HEPES and stained with Nonyl Acridine Orange (NAO; 0.1 mM; Invitrogen) for 30 min at 37 °C in a 5% CO_2_ incubator. The stained cells were washed twice with HEPES, and fluorescence was detected on an EVOS M5000 (Thermo Fisher).

### Measurement of Oxygen Consumption Respiratory

C2C12 cells were seeded (2 × 10^4^) in Agilent Seahorse XF24 cell culture microplates. The Agilent Seahorse XFe24 extracellular flux assay kit was used to calibrate the cells for 24 h prior to OCR measurement. After 24 h, the media was replaced with XF DMEM (pH 7.4 with 5 mM HEPES, 200 mM sodium bicarbonate, 4.5 g/L glucose, and 4 mM L-glutamine). The cells were incubated for 30 min in a non-CO_2_ incubator at 37 °C and treated with Mito-Stress chemical (1.5 μM oligomycin, 1 μM FCCP, 0.5 μM rotenone/antimycin A) from the Calibrated XFe24 extracellular flux assay kit.

### Transmission electron microscopy

C2C12 cells were harvested by scraping in PBS. The suspended cells were spun down and washed with PBS three times. The cell pellet was fixed with 0.1 M phosphate buffer containing 2% glutaraldehyde (pH 7.3) for 2 h at room temperature. Subsequently, the cells were treated with 2% OsO4 plus 3% potassium ferrocyanide in 0.1 M cacodylate buffer (pH 7.3) for 1 h at 4 °C in the dark. After dehydration in an ethanol and propylene oxide series, the cells were embedded in Epon 812 and polymerized using pure resin at 70 °C for two days. Ultrathin sections (70 nm) were created with an ultramicrotome (University UCT, Leica, Installed at Korea Basic Science Institute) and collected on 150-mesh copper grids. After staining with uranyl (5 min) and lead citrate (3 min), the sections were examined via transmission electron microscopy (TEM) at 120 kV.

### Ex vivo isometric tetanic force

Intact TA muscles were dissected from the hindlimbs of euthanized mice and mounted vertically between a force transducer (Model FT03, Glass Instruments, USA) in an organ bath with platinum electrodes and continuous perfusion with 95% O_2_ + 5% CO_2_-saturated Krebs-Ringer solution (118 mM NaCl, 4.75 mM KCl, 24.8 mM NaHCO3, 1.18 mM KH2PO4, 2.5 mM CaCl2 ∙ 2H20, 1.18 mM MgSO4, and 10 mM glucose). Optimal muscle stretch was determined by applying a single twitch at a supramaximal voltage (100 V for 1 ms) using a previously described protocol with slight modification^24,25^ at the length that generated the maximal twitch force. TA muscles were subjected to different force frequencies (tetani with increasing stimulation frequencies at 1–180 Hz every 500 ms with 1-min recovery intervals). All of the experiments were performed at 25 °C. Data acquisition and analysis were performed using LabChart Pro Software (version 8; AD Instruments, Pty Ltd.). Muscle wet weight was measured at the end of each experiment.

### Statistical analysis

All of the statistical analyses were performed using GraphPad Prism 10. All of the experiments were repeated at least three times. The data are presented as the mean ± standard deviation (SD). The specific statistical tests performed for each experiment are described in the relevant figure legends.

## Results

### Decreased cardiolipin levels result in age-related muscle loss

Aging is characterized by a substantial decline in mitochondrial functionality, and mitochondrial dysfunction in aged tissues is associated with a notable reduction in CL levels in the mitochondria^26–29^. To determine the role of CL in skeletal muscle aging (Fig. 1a), we first quantified CL levels in the skeletal muscle from young (4-month-old) and aged (22-month-old) mice. Compared with that in young muscles, the CL levels in old muscles were significantly lower (Fig. 1b). We subsequently measured the expression levels of genes involved in CL synthesis and remodeling in skeletal muscle and confirmed that Crls1 mRNA expression was significantly decreased in the skeletal muscle of old mice (Fig. 1c), including within the heart, diaphragm, TA muscle, and soleus muscle; *Crls1* expression was most significantly decreased in the TA muscle of the hind limb (Fig. 1d). We also examined the protein levels in muscle tissue, revealing that there was a significant difference in Crls1 expression in the QD and TA muscles but not in the Sol, GA, or EDL muscles between young and old mice (Fig. 1e). In the soleus muscle, there was no corresponding significant changes at the protein level (Supplementary Fig. 1). Concurrently, the protein levels of the mitochondrial complex proteins were not decreased in the older group. This finding suggested that the absence of atrophy in the soleus may be attributed to the stable expression of the Crls1 protein and the maintenance of mitochondrial complex protein levels. Considering that CL interacts with mitochondrial complexes^30^, a decrease in CL levels could induce a reduction in the expression of mitochondrial complex proteins in old muscles. Hence, we investigated whether a decrease in CL levels contributes to the downregulation of mitochondrial complex proteins in an age-dependent manner. To this end, we performed immunoblot analyses of mitochondrial complex proteins and Crls1 from the TA muscles of old mice, in which both Crls1 and mitochondrial complex proteins were strongly downregulated (Fig. 1f). These results suggest that the decreased CL levels in the aged mitochondria of old mice is caused by a decrease in Crls1 expression; this downregulation of CL can cause mitochondrial dysfunction.

### AAV9-shCrls1-mediated *Crls1* downregulation decreases muscle mass, force, and mitochondrial complex protein levels in young TA muscle

**Fig. 2:**
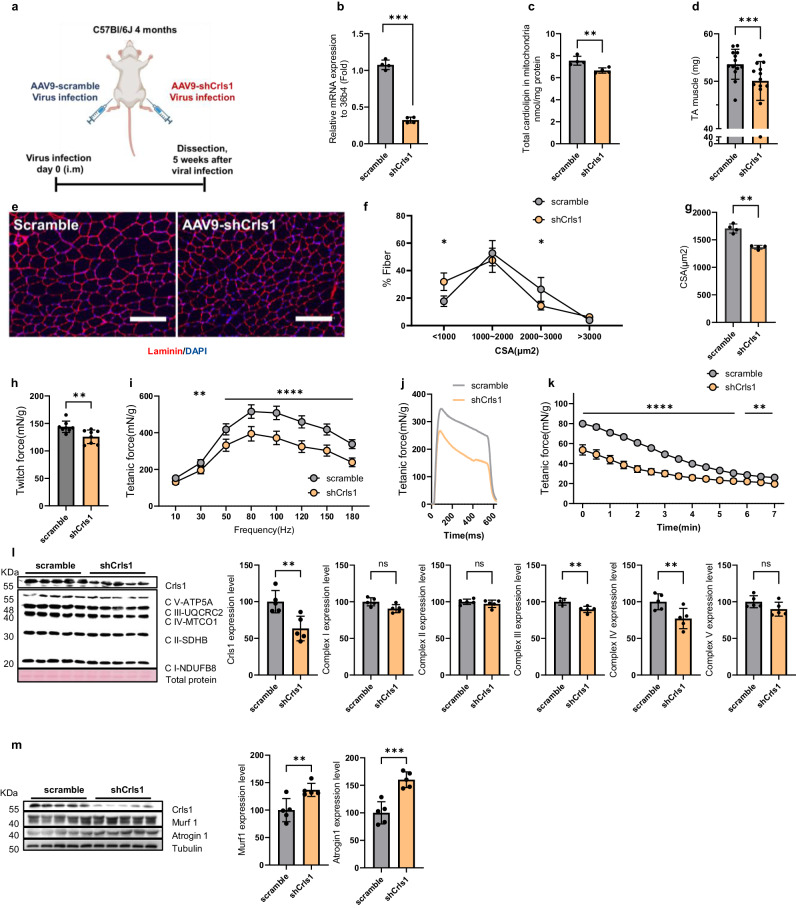
AAV9-shCrls1-mediated Crls1 decreased muscle mass, force and mitochondrial complex protein levels in young TA muscle. **a** Experimental schedule used to establish the AAV9-shCrls1-infected mouse model. **b** qPCR analysis of *Crls1* gene expression in scramble- and AAV9-shCrls1 virus-infected TA muscle (*n* = 4 per group). **c** Total cardiolipin levels in the mitochondria of scramble- and AAV9-shCrls1 virus-infected TA muscle (*n* = 4 per group). **d** Viral-infected TA muscle weight (*n* = 13 per group). **e** Histological analysis of virus-infected TA muscle. Tissue sections were stained with an anti-Laminin antibody. DAPI, blue; laminin, red. Scale bar in the left panel: 125 μm (the scramble and AAV9-shCrls1 groups were infected with AAV9 (virus diluted in PBS; total of 1×10^12^ GCs were injected by i.m.). Scale bar: 100 μm. **f** Myofiber cross-sectional area (CSA) fiber percentage in scramble- and AAV9-shCrls1 virus-infected TA muscle (*n* = 4 per group). **g** CSA fiber size mean in scramble- and AAV9-shCrls1 virus-infected TA muscle (n = 4 per group). **h** Measurement of the maximum twitch force at a supramaximal voltage (10 Hz) and 100 V for 1 ms (*n* = 8) in scramble- and AAV9-shCrls1 virus-infected TA muscle. **i** Frequency dependence (at 10–180 Hz, 100 V, 500 ms) of the average tetanic force in scramble- and AAV9-shCrls1 virus-infected TA muscle (*n* = 8 per group). **j** Tetanic force traces at 180 Hz for 500 ms in scramble- and AAV9-shCrls1 virus-infected TA muscle (*n* = 8 per group). **k** Fatigue indices were measured at 1 Hz and 100 V for 7 min (*n* = 8 per group). **l** Immunoblots of Crls1 and mitochondrial complex proteins in scramble- and AAV9-shCrls1 virus-infected TA muscle. The protein levels are relative to the total protein concentration (Ponceau) (*n* = 5 per group). Crls1 and mitochondrial complex protein expression levels relative to total protein in scramble- and AAV9-shCrls1 virus-infected TA muscle (*n* = 5 per group). **m** Immunoblotting of Crls1, Murf1, Atrogin1 and Tubulin in scramble- and AAV9-shCrls1 virus-infected TA muscle. Murf1 and Atrogin1 protein expression was normalized to tubulin (*n* = 5 per group). The data are presented as the mean ± standard deviation (SD) of *n* ≥ 3 independent experiments. *P* values were calculated using an unpaired Student’s t test. *P < 0.05; **P < 0.01; ***P < 0.001; n.s.: nonsignificant. C57Bl/6j young (4 to 5 mo.) mice were used.

To further investigate the impact of decreased CL abundance on the mitochondrial complex with aging, we knocked down Crls1 in young TA muscle using an AAV9 system—known to preferentially target myofibers and selectively suppress gene activity^31^ (Fig. 2a). After infection with AAV9-shCrls1, significant reductions in *Crls1* mRNA (Fig. 2b) and total CL (Fig. 2c) levels were observed in the TA muscles. Furthermore, the muscle weight of the AAV9-shCrls1 group was significantly lower than that of the AAV9-scramble group (Fig. 2d).

TA muscle sections were then subjected to anti-laminin immunofluorescence, and the size of the myofibrils was analyzed (Fig. 2e). Histological analysis of TA muscles revealed that a large proportion of fibers in the scramble group had an area <1000 μm², whereas most fibers in the AAV9-shCrls1 group had an area ranging from 1000 to <2000 μm², with a decreased mean fiber size (Figs. 2f, g). These findings indicate that *Crls1* affects muscle atrophy.

To evaluate the effect of *Crls1* on skeletal muscle function in intact muscles independent of other parameters, we compared the ex vivo contractile properties, force, and fatigability of young shCrls1- and scramble-treated TA muscles. The tetanic forces were decreased in the AAV9-shCrls1 group compared with those in the AAV9-scramble group (Figs. 2h–j). After repetitive stimulation at 1 Hz and 100 V for 7 min, which was used to cause fatigue, the tetanic force in the shCrls1 group was lower than that in the scramble group. Accordingly, the shCrls1 group appeared to be less sensitive to fatigue than the scramble group (Fig. 2k). Hence, downregulation of *Crls1* reduced the cross-sectional area (CSA) and strength of the TA muscle. Additionally, immunoblotting revealed significantly reduced levels of mitochondrial complex proteins in the shCrls1-treated TA muscles (Fig. 2l). To validate shCrls1-induced muscle atrophy, we examined the expression levels of Murf1 and Atrogin1, which are catabolic signaling markers, in sh-Crls1 muscles and scramble muscles. As shown in Fig. 2m, the levels of both Murf1 and Atrogin1 were increased in the shCrls-1-treated muscles, facilitating the breakdown of proteins within muscle fibers. Consistent with the findings of previous studies showing that downregulated *Cox7a1* and complex IV contribute to muscle atrophy^32,33^, we found that the reduction in complex IV levels due to *Crls1* knockdown resulted in muscle atrophy. Based on these findings, we hypothesize that Crls1 has a regulatory role in aging skeletal muscles, potentially influencing muscle regeneration and atrophy. To test this hypothesis, we investigated the association of *Crls1* with mitochondrial function and muscle atrophy by modulating *Crls1* expression.

### AAV9-mCrls1-mediated *Crls1* upregulation improves muscle mass, force, and mitochondrial complex protein levels in aging mice

**Fig. 3:**
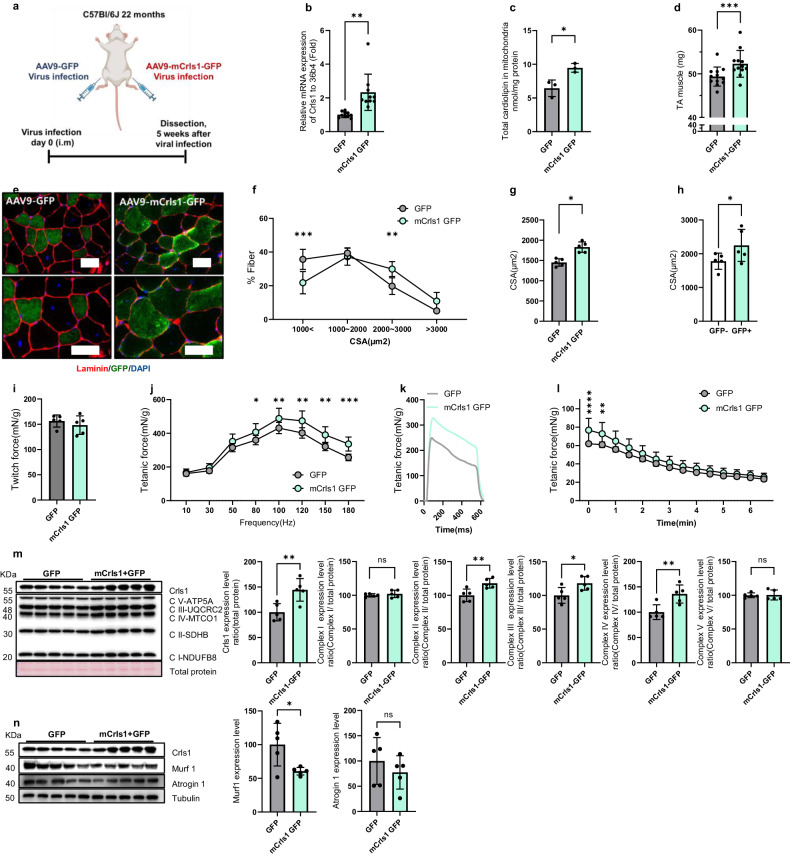
AAV9-mCrls1GFP-mediated Crls1 upregulation improved muscle mass, force and mitochondrial complex protein levels in aging mice. **a** Experimental schedule used to establish the AAV9-mCrls1 GFP-infected mouse model. **b** qPCR analysis of Crls1 gene expression in GFP- and mCrls1-GFP virus-infected TA muscle (*n* = 9 per group). **c** Total cardiolipin levels in the mitochondria of GFP- and mCrls1-GFP-infected TA muscle (*n* = 4 per group). **d** Viral-infected TA muscle weight (*n* = 13 per group). **e** Histological analysis of virus-infected TA muscle. Tissue sections were stained with anti-Laminin and anti-GFP antibodies. DAPI, blue; laminin, red; GFP, green. Scale bar left panel 50 µm(AAV9-GFP and AAV9-mCrls1-GFP group was infected by AAV9 (virus diluted in PBS virus total 5×1010 GC injected by i.m injection). (*n* = 5 per group). Scale bar: 50 μm. **f** Myofiber cross-sectional area (CSA) fiber percentage of GFP and mCrls1-GFP virus-infected TA muscle (*n* = 5 per group). **g** CSA fiber size mean in GFP- and mCrls1-GFP virus-infected TA muscle (*n* = 5 per group). **h** CSA fiber size-mean ratio of GFP- fibers to GFP+ fibers in AAV9-mCrls1-GFP virus-infected TA muscle (n = 5 per group). **i** Measurement of the maximum twitch force at a supramaximal voltage (10 Hz) and 100 V for 1 ms in GFP- and mCrls1-GFP virus-infected TA muscle (*n* = 5 per group). **j** Frequency dependence (at 10–200 Hz, 100 V, 500 ms) of the average tetanic force in GFP- and mCrls1-GFP virus-infected TA muscle (*n* = 5 per group). **k** Tetanic force traces at 180 Hz for 500 ms in GFP- and mCrls1-GFP virus-infected TA muscle (*n* = 5 per group). l Fatigue indices were measured at 1 Hz and 100 V for 6 min 30 sec (*n* = 5 per group). **m** Immunoblots of Crls1 and mitochondrial complex proteins in GFP- and mCrls1-GFP virus-infected TA muscle. The protein levels were calculated relative to the total protein concentration (Ponceau) (*n* = 5 per group). Crls1 and mitochondrial complex protein expression levels relative to total protein in GFP- and mCrls1-GFP virus-infected TA muscle (*n* = 5 per group). **n** Immunoblotting of Crls1, Murf1, Atrogin1 and Tubulin in GFP- and mCrls1-GFP virus-infected TA muscle. Murf1 and Atrogin1 protein expression was normalized to tubulin (*n* = 5 per group). The data are presented as the mean ± SEM of *n* ≥ 3 independent experiments. *P* values were calculated using an unpaired Student’s t test. *P < 0.05; **P < 0.01; ***P < 0.001; n.s.: nonsignificant. C57Bl/6j aged >22 mo. mice were used.

To assess whether *Crls1* increases the mass of aged TA muscles, AAV9-mCrls1-GFP was injected intramuscularly into mice (Fig. 3a). Five weeks after injection of the viral constructs, *Crls1* expression and total CL levels were increased in the mitochondria of AAV9-mCrls1-GFP-infected TA muscles compared to those in AAV9-GFP-infected TA muscles (Figs. 3b, c). The muscle weight of the AAV9-mCrls1-GFP group was significantly greater than that of the AAV9-GFP group (Fig. 3d). Histological analysis further revealed that the mean fiber size (1000 < and 2000–3000 μm^2^) was significantly greater in the AAV9-mCrs1-GFP group than in the AAV9-GFP group (Fig. 3e–g). Furthermore, compared with GFP-negative fibers, GFP-positive fibers exhibited an increased CSA in AAV9-mCrls1-GFP-infected TA muscles (Fig. 3h).

Similarly, we evaluated the functional consequences of overexpressing m*Crls1* in the context of aged skeletal muscle. The tetanic forces were greater in the AAV9-mCrls1-GFP group than in the AAV9-GFP group (Figs. 3i–k). Hence, upregulation of *Crls1* improved the CSA and strength of the TA muscle. After repetitive stimulation at 1 Hz and 100 V for 6 min, the tetanic force in the mCrls1-GFP group was greater than that in the GFP group. Accordingly, the mCrls1-GFP group appeared to be more sensitive to fatigue than the GFP group (Fig. 3l), suggesting that upregulation of Crls1 could increase the cross-sectional area (CSA) and strength of the TA muscle. Additionally, the levels of mitochondrial complex proteins and Crls1 were greater in the AAV9-mCrls1-GFP group than in the AAV9-GFP group (Fig. 3m). To validate the effects of Crls1 upregulation, we examined the expression levels of Murf1 and Atrogin1, which are catabolic signaling markers, in mCrls1-GFP and GFP muscle samples. As shown in Fig. 3n, the levels of both Murf1 and Atrogin1 were decreased in the upregulation group, which might inhibit the breakdown of proteins within muscle fibers. Taken together, these findings suggest that *Crls1* upregulation via AAV9-mCrls1-GFP in aged skeletal muscles improved muscle mass, fiber size, force and mitochondrial complex protein expression.

Next, we investigated whether the regulation of the Crls1 gene led to structural changes in the mitochondria of muscle tissue. Transmission electron microscopy (TEM) was utilized to observe mitochondria in TA muscle infected with scramble, shCrls1, GFP, or mCrls1-GFP (Supplementary Fig. 2a). The shCrls1 group exhibited an increased perimeter of mitochondria compared to that of the scramble group, while the mCrls1-GFP group demonstrated a decreased perimeter of mitochondria compared to that of the GFP group (Supplementary Fig. 2b). Additionally, the z-line length was shorter in the shCrls1 group than in the scramble group. Additionally, the mCrls1 group had an increased z-line length compared to that of the GFP group (Supplementary Fig. 2c). Overall, the TEM images revealed that restoration of the Crls1 protein in old muscles promoted healthier mitochondrial morphology, which was similar to what was found in young muscles.

### Mitochondrial *Crls1* and CL are essential for myogenesis in C2C12 cells

**Fig. 4:**
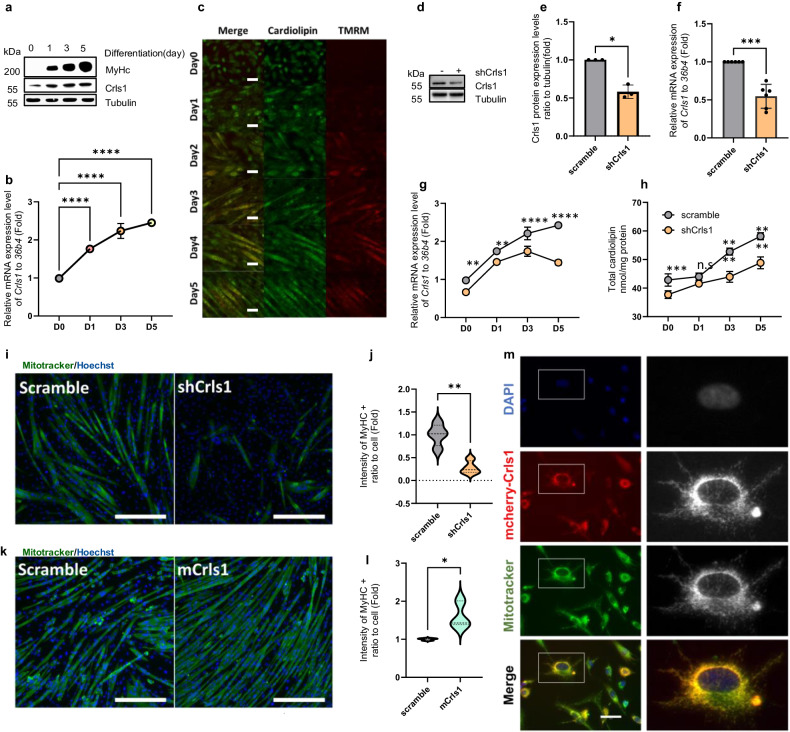
Mitochondrial *Crls1* and cardiolipin are essential for myogenesis in C2C12 cells. **a** Immunoblotting of whole-cell lysates from myoblasts and myotubes differentiated for 1, 3, and 5 days. **b** qPCR analysis of *Crls1* expression in myoblasts and myotubes differentiated for 1, 3, and 5 days (*n* = 3). **c** Live-cell staining of cardiolipin and mitochondria by NAO and TMRM in myoblasts and myotubes differentiated for 1–5 days. Scale bar: 50 μm. **d** Immunoblotting of whole-cell lysates from scramble- and shCrls1-transfected C2C12 cells. **e** CRLS1 protein expression relative to tubulin (*n* = 3). **f** qPCR analysis of *Crls1* gene expression in the *Crls1-*knockdown cell line (*n* = 6). **g** qPCR analysis of *Crls1* gene expression in the Crls1-knockdown cell line during myogenesis (*n* = 3). **h** Total cardiolipin levels in myoblasts and myotubes differentiated for 1, 3, and 5 days (*n* = 4). **i** Representative images of myosin heavy chain (MyHC) staining of C2C12-derived myotubes at myogenic differentiation Day 5 after infection with scramble or shCrls1. 4′,6-Diamidino-2-phenylindole (DAPI) was used as a nuclear counterstain. Scale bar: 100 μm. **j** Measurement of the green fluorescence intensity in Myhc-stained myotubes. (*n* = 4). **k** Representative images of myosin heavy chain (MyHC) staining of C2C12-derived myotubes at myogenic differentiation Day 5 after infection with scramble or shCrls1. Scale bar: 100 μm in length. **l** Measurement of the green fluorescence intensity in Myhc-stained myotubes. (*n* = 3). **m** MitoTracker green staining of Crls1-mCherry-transfected C2C12 cells. Blue: Hoechst 33342, red: mCherry-Crls1, green: MitoTracker-green FM, yellow: merged. (right) Enlarged view of the boxed areas. Scale bar: 50 μm. The data are presented as the mean ± SD of *n* ≥ 3 independent experiments. *P* values were calculated using an unpaired Student’s *t* test. *P < 0.05; **P < 0.01; ***P < 0.001; paired *t* test (**e** and **f**). n.s.: nonsignificant. scramble: untargeted shRNA-Lentivirus-infected cell line; shCrls1: *Crls1*-targeting shRNA-lentivirus-infected cell line.

In addition to considering the decrease in muscle regeneration that occurs with age, we investigated the effects of manipulating the Crls1 gene on muscle cell myogenesis in vitro. To confirm the effects of *Crls1* modulation on myogenesis in vitro in C2C12 cells, we examined changes in *Crls1* expression patterns and mitochondrial OXPHOS complex proteins after C2C12 cells were differentiated for 1, 3, and 5 days (Fig. 4a). With the progression of differentiation, a noticeable increase in *Crls1* mRNA levels was observed (Fig. 4b), as was a concomitant increase in CL levels and mitochondrial mass (Fig. 4c).

To determine the impact of *Crls1* downregulation on myogenesis, we constructed a *Crls1-*knockdown cell line using *Crls1*-targeted shRNA and observed a significant decrease in CL protein abundance (Figs. 4d, e). Consistent with the decrease in protein levels, *Crls1* mRNA expression decreased during myogenesis (Figs. 4f, g). Moreover, a 10% decrease in CL levels was observed during differentiation in the *Crls1* knockdown cell line compared to the control cells (Fig. 4h). Furthermore, the expression levels of the myogenesis markers Myh7, Mhy2, Myh4 and Myh1 during differentiation were significantly decreased in the *Crls1* knockdown cell line (Supplementary Fig. 3). These results were consistent with the decreased expression of myogenesis-specific genes and reduced MyHC staining observed 5 days after *Crls1* knockdown (Figs. 4i, j). Conversely, overexpression of *Crls1* resulted in increased MyHC-positivity (Figs. 4k, l), demonstrating that *Crls1* plays a critical role in myogenesis. We also observed the colocalization of Crls1-mCherry and MitoTracker (Fig. 4m), confirming that CL regulation by *Crls1* during myogenesis affects muscle cell differentiation.

### shCrls1-mediated *Crls1* downregulation induces mitochondrial dysfunction via mitochondrial structure disorder

**Fig. 5:**
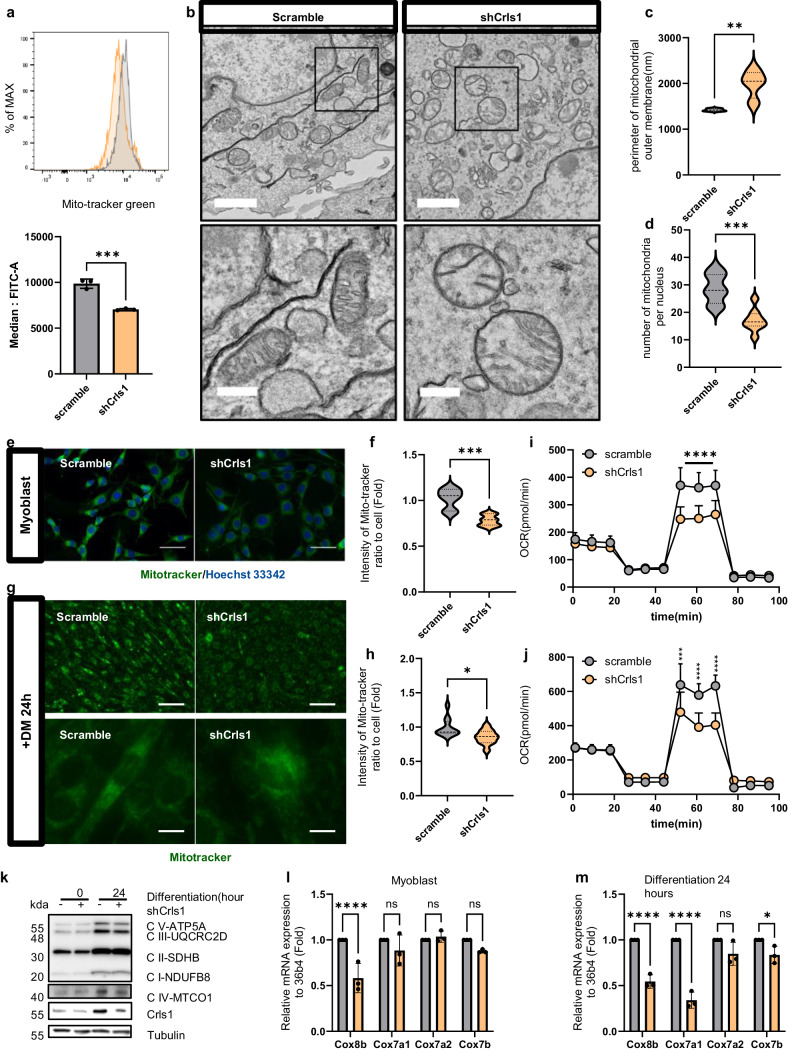
*Crls1* affects mitochondrial structure, leading to the modulation of mitochondrial activity. **a** Analysis of mitochondrial mass using flow cytometry. MitoTracker Green staining of myoblasts (gray graph: scramble-infected cell line; yellow: sh-Crls1-infected cell line). Bottom graph: median FITC (green). **b** Analysis of mitochondrial structure via transmission electron microscopy (left top and bottom panels: infected with scramble; right top and bottom panels: infected with shCrls1; top scale bar: 1 μm; bottom scale bar: 300 nm). **c** Mitochondrial outer membrane parameter. **d** Number of mitochondria per nucleus. **e** Live cell image stained with MitoTracker (left panel: scramble-infected cell line; right panel: shCrls1-infected cell line). Scale bar: 50 μm. **f** Measurement of the green fluorescence intensity in MitoTracker-stained myoblasts. (*n* = 7). **g** Live cell image stained with MitoTracker in the differentiated C2C12 cell line for 24 h in differentiation medium (left panel: scramble-infected cell line; right panel: shCrls1-infected cell line). Top scale bar: 100 μm, bottom scale bar: 12.5 μm. **h** Green fluorescence intensity of differentiated cells treated with DM for 24 **h** (*n* = 11). **i** Oxygen consumption rate (OCR) in myoblasts (*n* = 10). **j** OCRs of differentiated C2C12 cells cultured in differentiation medium for 24 h (*n* = 4). **k** Immunoblotting of whole-cell lysates from scramble and shCrls1 myoblasts differentiated for 0 and 24 h. **l** qPCR analysis of the mitochondrial complex 4 gene from scramble and shCrls1 myoblasts (*n* = 3). m qPCR analysis of the mitochondrial complex 4 gene from scramble- and shCrls1-differentiated myotubes on Day 1 (*n* = 3). The graphs present the mean ± SD of *n* ≥ 3 independent experiments*. P* values were calculated using an unpaired Student’s *t* test. *P < 0.05; **P < 0.01; ***P < 0.001; paired *t* test (**a**). n.s.: nonsignificant. scramble: untargeted shRNA-lentivirus-infected cell line; shCrls1: *Crls1* target shRNA-lentivirus-infected cell line.

Next, we focused on comprehensively evaluating mitochondrial activity, cristae structure, the oxygen consumption rate (OCR), and the expression of OXPHOS complex IV genes. Using flow cytometry, we confirmed a significant decrease in mitochondrial mass in shCrls1 cells (Fig. 5a), demonstrating the effect of *Crls1* knockdown on myoblasts. Specifically, TEM examination of the mitochondrial structure revealed a decreased number of mitochondria in shCrls1 cells and increased mitochondrial outer membrane parameters (Fig. 5b–d). The reduction in mitochondrial mass was further verified using fluorescence microscopy with MitoTracker, which revealed a noticeable decrease in intensity (Fig. 5e, f). Additionally, compared with control cells, shCrls1 cells exhibited delayed differentiation and decreased MitoTracker staining after 24 h (Fig. 5g, h). Additionally, quantification of the OCR confirmed decreased mitochondrial activity in *Crls1* knockdown cells, as indicated by a significant reduction in the maximum OCR compared to that in the control group (Fig. 5i, Supplementary Fig. 4a, b). A substantial decrease in the maximum OCR was observed in myoblasts and myotubes with Crls1 knockdown (Fig. 5j, Supplementary Fig. 4c, d). Additionally, ATP production was lower in shCrls1 knockdown cell lines than in scramble cell lines (Supplementary Fig. 4e).

The abundance of OXPHOS complex proteins and *Crls1* was significantly reduced (Fig. 5k). Notably, the protein levels of complex IV gradually increased in myotubes compared to those in myoblasts; this increase was not observed in *Crls1-*knockdown cells during differentiation. We further analyzed the mRNA expression levels of complex IV genes during differentiation following *Crls1* knockdown (Fig. 5l). The expression of these genes was downregulated following *Crls1* knockdown compared to that in the scramble group. Additionally, mRNA analysis of cells differentiated for 1 day consistently revealed reduced expression levels of the OXPHOS complex IV genes in the *Crls1* knockdown cell line over time (Fig. 5m).

Overall, the downregulation of *Crls1* during myogenesis decreased mitochondrial respiration in myoblasts and myotubes and suppressed the increase in mitochondrial protein and gene expression levels during differentiation. These findings suggest that *Crls1* knockdown induces mitochondrial dysfunction, as indicated by the downregulated expression of mitochondrial complex proteins during myogenesis. Based on these findings, we performed muscle regeneration experiments using a murine skeletal muscle injury model.

### Restoration of *Crls1* promotes muscle regeneration in the TA muscles of young and old mice

**Fig. 6:**
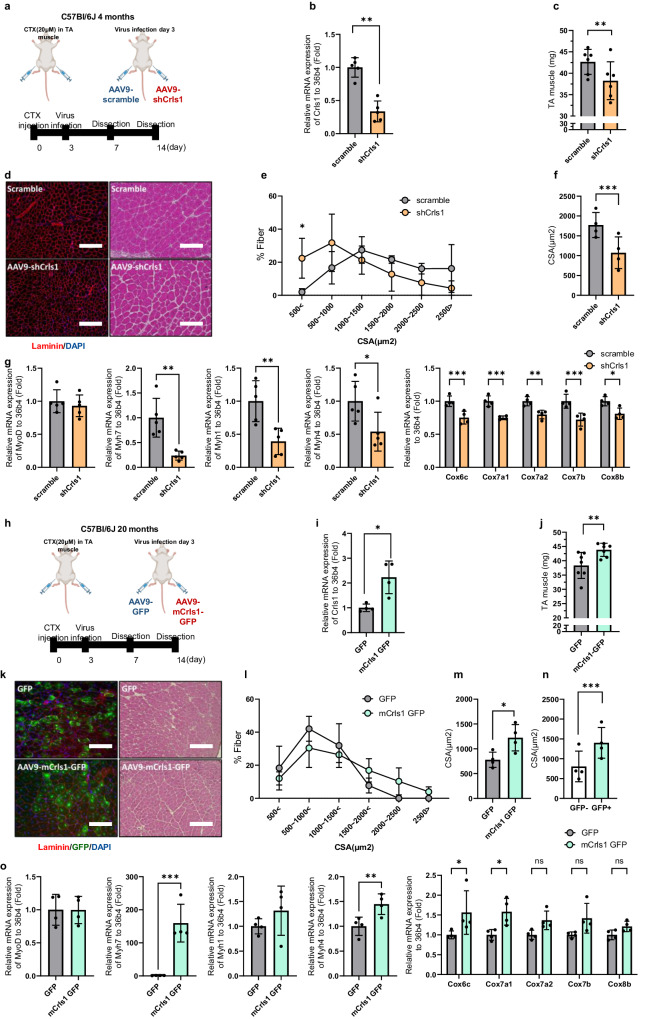
Restoration of Crls1 promotes muscle regeneration in the TA muscles of young and old mice. **a** Experimental schedule for establishing the virus-infected mouse and regeneration mouse models. **b** qPCR analysis of Crls1 genes in virus-infected and regenerated young TA muscle (*n* = 5 per group). **c** Viral infection and regenerated young TA muscle weight (*n* = 6 per group). **d** Histological analysis. Tissue sections from virus-infected and regenerated TA muscle was stained with anti-Laminin and H&E. DAPI, blue; laminin, red. Scale bar: 50 μm (AAV9-scramble and AAV9-shCrls1 groups were infected with AAV9 (virus diluted in PBS; total of 1×10^12^ GCs were injected i.m.). **e** Myofiber cross-sectional area (CSA) fiber percentage in scramble- and AAV9-shCrls1 virus-infected TA muscle (*n* = 4 per group). **f** Mean CSA fibers in the TA muscle (*n* = 4 per group). **g** qPCR analysis of the MyoD, Myh7, Myh4, Myh1, Cox6b, Cox7a1, Cox7a2, Cox7b and Cox8b genes in virus-infected and regenerated young TA muscle (*n* = 4 per group). **h** Experimental schedule for establishing a virus-infected mouse and a regeneration old mouse model. **i** qPCR analysis of Crls1 gene expression in virus-infected old TA muscle (*n* = 4 per group). **j** Viral infection and regenerated old TA muscle weight (*n* = 7 per group). **k** Histological analysis of virus-infected and regenerated TA muscle tissue sections stained with anti-Laminin and H&E. DAPI, blue; laminin, red. Scale bar: 125 µm (AAV9-GFP and AAV9-mCrls1-GFP groups) were infected with AAV9 (virus diluted in PBS; total 5×10^9^ GCs were injected i.m.). Sectional tissue was stained with an anti-laminin antibody from AAV9-mCrls1-GFP-infected old tissue. Laminin; red, GFP; green, DAPI; blue. Scale bar = 125 µm. **l** Myofiber cross-sectional area (CSA) fiber percentage in AAV-GFP- and AAV9-mCrls1-GFP-infected TA muscle (*n* = 4 per group). **m** Mean CSA fibers in the TA muscle (*n* = 4 per group). **n** CSA fiber size mean of GFP^-^ fibers and GFP^+^ fibers in AAV9-mCrls1-GFP virus-infected and regeneration TA muscle (*n* = 4 per group). **o** qPCR analysis of MyoD, Myh7, Myh4, Myh1, Cox6b, Cox7a1, Cox7a2, Cox7b and Cox8b in virus-infected old TA muscle (*n* = 4 per group). The data are presented as the mean ± SD of *n* ≥ 3 independent experiments. *P* values were calculated using an unpaired Student’s t test. *P < 0.05; **P < 0.01; ***P < 0.001; n.s.: nonsignificant. C57Bl/6j young (4 to 5 mo.) mice were used. C57Bl/6j mice aged >20 mo. mice were used.

Next, we investigated the effects of Crls1 regulation on regeneration using a CTX-induced skeletal muscle injury model (Fig. 6a). Fourteen days after injection of AAV9-shCrls1 into the TA muscles of mice, the *Crls1* expression and muscle weight were lower than those in the AAV9-scramble group (Figs. 6b, c). Histological analysis of TA muscle samples collected 14 days postinjury revealed a notable increase in the percentage of fibers with an average size smaller than 500 μm² in the AAV9-shCrls1 group compared to that in the scramble group (Figs. 6d, e). Nevertheless, the administration of AAV9-mCrls1 occurred at the peak of myoblast proliferation, a phase which remains unaffected by the dynamics of myoblast growth and is identified three days post-injury (Supplementary Fig. 5). Additionally, compared with those in the scramble group, the mean cross-sectional area (CSA) in the shCrls1 group was reduced (Fig. 6f). Histologically, we confirmed that Crls1 downregulation inhibited regeneration, and additionally, we compared the gene expression levels of muscle fiber markers and mitochondrial complex 4 genes during the process of regeneration (Fig. 6g). Consequently, the expression levels of Myh7 and Myh1 were significantly lower in the shCrls1 group than in the scramble group.

In contrast, to determine the gain-of-function effect of *Crls1*, we constructed an AAV9-mCrls1-GFP virus to improve muscle regeneration in aged TA muscles (Fig. 6h). TA muscles injected with AAV9-mCrls1-GFP post-CTX injury on Day 14 presented increased *Crls1* expression and muscle weight compared to those of the AAV9-scramble group (Fig. 6i, j). According to the histological analysis of TA muscle samples obtained 14 days postinjury, the average cross-sectional area (CSA) was increased in the AAV9-mCrls1 group compared to the AAV9-GFP group (Fig. 6k–m). Compared with GFP- fibers, GFP+ fibers also exhibited increased mean CSA fiber sizes in AAV9-mCrls1-GFP-infected TA muscles (Fig. 6n). The overexpression of Crls1 was histologically confirmed to improve regeneration, and additionally, we compared the gene expression levels of muscle fiber markers and mitochondrial complex 4 genes during the process of regeneration (Fig. 6o). In contrast to the results of the loss-of-function experiments above, the expression levels of Myh7 were markedly greater in the mCrls1-GFP group than in the GFP group (Fig. 6o). These data demonstrated that *Crls1* overexpression improved skeletal muscle regeneration in old mice. Taken together, our results provide novel insights into the impact of age-dependent *Crls1* loss on muscle health and suggest potential therapeutic targets for skeletal muscle myopathy.

## Discussion

A decrease in body muscle mass of 50–80% in elderly individuals is associated with decreased strength and increased mortality^34^. Mitochondrial quality control is important for maintaining muscle function and mass. According to a recent study, high concentrations of CL in mitochondria not only promote membrane curvature in cristae but are also essential for efficient electron transport and OXPHOS^16^. Decreased CL levels in skeletal muscle have been associated with mitochondrial defects and compromised energy efficiency^35^.

Here, we found that age-dependent mitochondrial dysfunction is linked not only to a diminished ability to generate ATP but also to the activation of pathways that result in irreversible cell loss. Our data corroborated the presence of age-related sarcopenia, particularly focusing on CL levels in the TA muscle, which were reduced in an age-dependent manner. Specifically, we detected a reduction in CL levels in 22-month-old mice with concomitant decreases in *Crls1* expression, suggesting that this change might be strongly linked to TA muscle loss in old mice. Moreover, the downregulation of *Crls1* in the TA muscles of young mice resulted in a reduction in myofiber number, muscle mass, and the expression of mitochondrial complex IV mRNA and protein. Moreover, the upregulation of *Crls1* in the TA muscles of old mice induced the opposite effects. Additionally, *Crls1* knockdown in C2C12 cells decreased myogenesis and the mitochondrial OCR, which correlated with reduced expression levels of mitochondrial complex IV genes and mitochondrial cristae destruction. Hence, *Crls1* expression and CL synthesis are associated with skeletal muscle regeneration. Collectively, these findings suggest that *Crls1* plays a critical role in preserving mitochondrial membrane organization through CL and enhancing mitochondrial function during muscle damage. A previous study reported that muscle hypertrophy is induced in plantar muscle tissue as a result of overload, and the CL level and complex IV protein expression increase^36^. Moreover, deleterious variants of *CRLS1* in humans cause CL deficiency and autosomal recessive multilineage mitochondrial disease^37^. Consistent with these reports, our data showed that the downregulation of *Crls1* decreased mitochondrial complex IV expression in old mice.

Knockdown or overexpression of *Crls1* showed substantial potential for promoting muscle regeneration in C2C12 cells. To validate these findings in vivo, we generated a CTX injury model in young and old mice with *Crls1* knockdown or overexpression. In *Crls1*-knockdown mice, myogenesis-related gene expression was reduced, regenerative capacity was diminished, and the CSA of muscle fibers was decreased in the injured muscles. Conversely, overexpression of *Crls1* in mice increased mitochondrial complex IV gene expression, enhanced muscle force, and increased muscle fiber CSA in the injured muscles. These findings suggest that mitochondrial structure and function can be regulated by modulating *Crls1* expression in mouse muscle tissue. Indeed, enhancing mitochondrial function through the upregulation of *Crls1* is useful for improving muscle atrophy and regeneration in aged skeletal muscles.

Our data suggest that *Crls1* downregulation suppresses OXPHOS capacity during myogenesis, which is a fundamental component of energy metabolism. Although *Crls1* in muscles enhances mitochondrial OXPHOS and muscle regeneration, thus reducing muscle atrophy, functional concerns regarding *Crls1* expression require further elucidation in different tissues. Future studies should precisely address the metabolic changes that occur through *Crls1* regulation and elucidate whether the regulation of metabolic shifts by *Crls1* affects muscle type.

In conclusion, we provide evidence that age-associated muscle damage is caused by reduced *Crls1* levels. Conversely, *Crls1* upregulation can potentially facilitate CL synthesis, modulating the structure of mitochondrial cristae and enhancing mitochondrial function through the expression of complex IV. Although the specific signaling pathway remains elusive, *Crls1* has promising potential for ameliorating sarcopenia by influencing muscle regeneration processes. Conceptually, these findings suggest that *Crls1* in aged muscles can be therapeutically manipulated to improve muscle regeneration and decrease muscle atrophy.

### Supplementary information

Supplementary Materials for Age-dependent loss.

## Data Availability

All of the original data are available from the authors without any restrictions.

## References

[1] Frontera WR, Ochala J (2015). Skeletal muscle: a brief review of structure and function. Calcif. Tissue Int.

[2] Rolland Y (2008). Sarcopenia: its assessment, etiology, pathogenesis, consequences and future perspectives. J. Nutr. Health Aging.

[3] von Haehling S, Morley JE, Anker SD (2010). An overview of sarcopenia: facts and numbers on prevalence and clinical impact. J. Cachexia Sarcopenia Muscle.

[4] Cohen S, Nathan JA, Goldberg AL (2015). Muscle wasting in disease: molecular mechanisms and promising therapies. Nat. Rev. Drug Discov..

[5] Leduc-Gaudet JP, Hussain SNA, Barreiro E, Gouspillou G (2021). Mitochondrial Dynamics and Mitophagy in Skeletal Muscle Health and Aging. Int J. Mol. Sci..

[6] Lee CE, McArdle A, Griffiths RD (2007). The role of hormones, cytokines and heat shock proteins during age-related muscle loss. Clin. Nutr..

[7] Larsson L (2019). Sarcopenia: Aging-Related Loss of Muscle Mass and Function. Physiol. Rev..

[8] Yin L (2021). Skeletal muscle atrophy: From mechanisms to treatments. Pharm. Res.

[9] Sartori R, Romanello V, Sandri M (2021). Mechanisms of muscle atrophy and hypertrophy: implications in health and disease. Nat. Commun..

[10] Abrigo J, Simon F, Cabrera D, Vilos C, Cabello-Verrugio C (2019). Mitochondrial Dysfunction in Skeletal Muscle Pathologies. Curr. Protein Pept. Sci..

[11] Alston CL, Rocha MC, Lax NZ, Turnbull DM, Taylor RW (2017). The genetics and pathology of mitochondrial disease. J. Pathol..

[12] Hong X (2022). Mitochondrial dynamics maintain muscle stem cell regenerative competence throughout adult life by regulating metabolism and mitophagy. Cell Stem Cell.

[13] Kojima R (2019). Maintenance of Cardiolipin and Crista Structure Requires Cooperative Functions of Mitochondrial Dynamics and Phospholipid Transport. Cell Rep..

[14] Funai K, Summers SA, Rutter J (2020). Reign in the membrane: How common lipids govern mitochondrial function. Curr. Opin. Cell Biol..

[15] Pennington ER, Funai K, Brown DA, Shaikh SR (2019). The role of cardiolipin concentration and acyl chain composition on mitochondrial inner membrane molecular organization and function. Biochim Biophys. Acta Mol. Cell Biol. Lipids.

[16] Dudek J (2017). Role of Cardiolipin in Mitochondrial Signaling Pathways. Front Cell Dev. Biol..

[17] Ren M, Phoon CK, Schlame M (2014). Metabolism and function of mitochondrial cardiolipin. Prog. Lipid Res.

[18] Claypool SM, Oktay Y, Boontheung P, Loo JA, Koehler CM (2008). Cardiolipin defines the interactome of the major ADP/ATP carrier protein of the mitochondrial inner membrane. J. Cell Biol..

[19] Fry M, Green DE (1981). Cardiolipin requirement for electron transfer in complex I and III of the mitochondrial respiratory chain. J. Biol. Chem..

[20] Robinson NC (1993). Functional binding of cardiolipin to cytochrome c oxidase. J. Bioenerg. Biomembr..

[21] Lange C, Nett JH, Trumpower BL, Hunte C (2001). Specific roles of protein-phospholipid interactions in the yeast cytochrome bc1 complex structure. EMBO J..

[22] Ozawa T, Tanaka M, Wakabayashi T (1982). Crystallization of mitochondrial cytochrome oxidase. Proc. Natl Acad. Sci. USA.

[23] Lu YW, Claypool SM (2015). Disorders of phospholipid metabolism: an emerging class of mitochondrial disease due to defects in nuclear genes. Front Genet.

[24] Ursu D (2001). Excitation-contraction coupling in skeletal muscle of a mouse lacking the dihydropyridine receptor subunit gamma1. J. Physiol..

[25] Dayal A (2017). The Ca(2+) influx through the mammalian skeletal muscle dihydropyridine receptor is irrelevant for muscle performance. Nat. Commun..

[26] Petrosillo G, Matera M, Casanova G, Ruggiero FM, Paradies G (2008). Mitochondrial dysfunction in rat brain with aging Involvement of complex I, reactive oxygen species and cardiolipin. Neurochem Int.

[27] Petrosillo G, De Benedictis V, Ruggiero FM, Paradies G (2013). Decline in cytochrome c oxidase activity in rat-brain mitochondria with aging. Role of peroxidized cardiolipin and beneficial effect of melatonin. J. Bioenerg. Biomembr..

[28] Lesnefsky EJ, Hoppel CL (2008). Cardiolipin as an oxidative target in cardiac mitochondria in the aged rat. Biochim Biophys. Acta.

[29] Paradies G, Ruggiero FM, Petrosillo G, Quagliariello E (1997). Age-dependent decline in the cytochrome c oxidase activity in rat heart mitochondria: role of cardiolipin. FEBS Lett..

[30] Paradies G, Paradies V, Ruggiero FM, Petrosillo G (2019). Role of Cardiolipin in Mitochondrial Function and Dynamics in Health and Disease: Molecular and Pharmacological Aspects. Cells.

[31] Zincarelli C, Soltys S, Rengo G, Rabinowitz JE (2008). Analysis of AAV serotypes 1-9 mediated gene expression and tropism in mice after systemic injection. Mol. Ther..

[32] Brown JL (2017). Mitochondrial degeneration precedes the development of muscle atrophy in progression of cancer cachexia in tumour-bearing mice. J. Cachexia Sarcopenia Muscle.

[33] Ronn T (2008). Age influences DNA methylation and gene expression of COX7A1 in human skeletal muscle. Diabetologia.

[34] Fielding RA (2011). Sarcopenia: an undiagnosed condition in older adults. Current consensus definition: prevalence, etiology, and consequences. International working group on sarcopenia. J. Am. Med Dir. Assoc..

[35] Prola A (2021). Cardiolipin content controls mitochondrial coupling and energetic efficiency in muscle. Sci. Adv..

[36] Fajardo VA, Mikhaeil JS, Leveille CF, Saint C, LeBlanc PJ (2017). Cardiolipin content, linoleic acid composition, and tafazzin expression in response to skeletal muscle overload and unload stimuli. Sci. Rep..

[37] Lee RG (2022). Deleterious variants in CRLS1 lead to cardiolipin deficiency and cause an autosomal recessive multi-system mitochondrial disease. Hum. Mol. Genet.

