# Author Correction: Age-dependent loss of *Crls1* causes myopathy and skeletal muscle regeneration failure

**DOI:** 10.1038/s12276-024-01238-7

**Published:** 2024-04-24

**Authors:** Youngbum Yoo, MyeongHoon Yeon, Won-Kyung Kim, Hyeon-Bin Shin, Seung-Min Lee, Mee-Sup Yoon, Hyunju Ro, Young-Kyo Seo

**Affiliations:** 1 Aging Convergence Research Center, Korea Research Institute of Bioscience and Biotechnology (KRIBB), Daejeon, 34141 Republic of Korea; 2 Department of Biological Sciences, College of Bioscience and Biotechnology, Chungnam National University, Daejeon, 34134 Republic of Korea; 3 Biomolecular Science, KRIBB School of Bioscience, Korea University of Science and Technology (UST), Daejeon, 34141 Korea; 4 Department of Molecular Medicine, College of Medicine, Gachon University College of Medicine, Incheon, 21999 Republic of Korea; 5 Department of Health Sciences and Technology, GAIHST, Gachon University, Incheon, 21999 Republic of Korea; 6 School of Medicine, Sungkyunkwan University, Suwon, 16419 Republic of Korea

**Keywords:** Cell biology, Gene expression analysis, Predictive markers

Correction to: *Experimental & Molecular Medicine* 10.1038/s12276-024-01199-x, published online 01 April 2024

In the Acknowledgements section of this article the grant numbers relating to NRF given for Young-Kyo Seo was incorrectly given as OGM6512312 and should have been 2022R1A2C109179013, to NST given for Young-Kyo Seo was incorrectly given as NTC0132312 and should have been CRC22012-200, and to Ministry of Health and Welfare of Korea for Young-Kyo Seo was incorrectly given as BGC1502312 and should have been HV22C012800.

The original article has been corrected.

