# Supplementary material for: Age-dependent loss of Crls1 causes myopathy and skeletal muscle regeneration failure
Source: Exp Mol Med. 2024 Apr 1;56(4):922–34. doi: 10.1038/s12276-024-01199-x (PMC11059380; doi:10.1038/s12276-024-01199-x)
Supplement: Supplementary file 1 — Supplementary Materials for Age-dependent loss. [file 12276_2024_1199_MOESM1_ESM.docx]

Supplementary Materials for

**Age-dependent loss of Crls1 causes myopathy and skeletal muscle regeneration failure**

Youngbum Yoo^1, 2^, MyeongHoon Yeon^1^, Won-Kyung Kim^1,3^, Hyeon-Bin Shin^1,3^, Seung-Min Lee^1^, Mee-Sup Yoon^4^, 5, Hyunju Ro^2^ and Young-Kyo Seo^1,3,6,^ ^#^

^1^Aging Convergence Research Center, Korea Research Institute of Bioscience and Biotechnology (KRIBB), Daejeon 34141, Republic of Korea

^2^Department of Biological Sciences, College of Bioscience and Biotechnology, Chungnam National University, Daejeon 34134, Republic of Korea

^3^Biomolecular Science, KRIBB School of Bioscience, Korea University of Science and Technology (UST), Daejeon 34141, Korea

^4^Department of Molecular Medicine, College of Medicine, Gachon University College of Medicine, Incheon 21999, Republic of Korea.

^5^Department of Health Sciences and Technology, GAIHST, Gachon University, Incheon 21999, Republic of Korea

^6^School of Medicine, Sungkyunkwan University, Suwon 16419, Republic of Korea

# Corresponding author: Y.K. Seo ([ykseo@kribb.re.kr](mailto:ykseo@kribb.re.kr))

**This PDF file includes:**

Sup Fig1 to 5

**Supplementary Materials and Methods**

**Animal care**

Young (3 to 5-month-old) and old (22-month-old) C57BL/6J mice were purchased from the Laboratory Animal Resource Center (Korea Research Institute of Bioscience and Biotechnology (KRIBB)). The animal experiments were approved by KRIBB-IACUC. All mice were provided a standard laboratory diet (3.1 kcal/g) purchased from Damul Science (Daejeon, Korea). Complete randomization was applied for group assignment and experimental selection.

**Immunoblotting**

Protein was extracted from the whole cell lysate and tissues with RIPA lysis buffer (50 mM Tris/HCl, pH 8.0, 150 mM NaCl, 1% NP-40, and 0.1% SDS) containing a Protease and Phosphatase inhibitor cocktail (Roche). After homogenization using FastPrep24TM (MP Biomedicals™) or sonication (Fangxu), cell and tissue lysates were incubated on ice for 30 min and centrifuged at 14,000 ×g for 20 min at 4 °C. The supernatant was collected, and protein estimation was performed using the Pierce™ Rapid Gold BCA Protein Assay Kit (Thermo Fisher) before addition of 5× SDS sample buffer containing 2-mercaptoethanol (Bio Rad). Total protein lysate (10–20 mg) was resolved on SDS-polyacrylamide gel according to the standard procedure at 25 mA per gel and blotted onto a PVDF 0.2 μm membrane (Bio rad) via the Mini Trans-blot module (Bio-Rad) at a constant voltage (250 mA) for 2 h. After blocking with 3% bovine serum albumin in Tris-buffered saline with 0.05% Tween 20 (TBS-T) for 1 h, the membrane was incubated overnight in 5% BSA/TBS-T with primary antibodies (Table S1). The membranes were then incubated with secondary specific antibodies (Table. S1). Membranes were scanned using the Luminograph II (ATTOKorea).

**Hematoxylin and Eosin staining**

Frozen muscle tissues were embedded in optimal cutting temperature compound and were sectioned (10 μm) using a cryostat (Leica CM1520). Sectional tissue was fixed by 4%PFA for 10min at RT. washing with PBS, hematoxylin staining was followed by washing with ddH2O, and after eosin staining, washing was performed with 70% EtOH, 80%, 90%, and 95%. Xylene was used to remove ethanol. Stained Tissue was mounted by mount solution (Fisher). Stained Tissue was detected by EVOS M5000 (Thermo Fisher).

**Transmission electron microscopy**

The skeletal muscle tissue was extracted from mice and subsequently sectioned into 1 mm3 pieces. C2C12 cells were harvested using a scraper in phosphate-buffered saline (PBS). The suspended cells underwent three PBS washes. Following this, both the cell pellets and the segmented skeletal muscle tissues were fixed in a 0.1M phosphate buffer containing 2% glutaraldehyde (pH 7.3) for a duration of 2 hours at room temperature. The sample then treated with 2% OsO4 and 3% potassium ferrocyanide dissolved in 0.1 M cacodylate buffer (pH 7.3) for 1 hour at 4°C in the dark. Cells were embedded in Epon 812 after dehydration in an ethanol and propylene oxide series and polymerized using pure resin at 70 °C for two days. Ultrathin sections (70 nm) were created with an ultra-microtome (ULTRACUT UCT, LEICA, Installed at Korea Basic Science Institute) and collected on 150-mesh copper grids. After staining with uranyless (5 min) and lead citrate (3 min), the sections were examined by transmission electron microcopy (TEM) at 120 kV.

**Ex vivo isometric tetanic force**

Intact TA muscles were dissected from the hindlimb of euthanized mice and mounted vertically between a force transducer (Model FT03, Glass Instruments, USA) in an organ bath with platinum electrodes and continuous perfusion with 95% O2 + 5% CO2-saturated Krebs-Ringer solution (118 mM NaCl, 4.75 mM KCl, 24.8 mM NaHCO3, 1.18 mM KH2PO4, 2.5 mM CaCl2∙2H20, 1.18 mM MgSO4, and 10 mM glucose). Optimal muscle stretch was determined by applying a single twitch at supramaximal voltage (100 V for 1 ms) using a previously described protocol with slight modification24,25 at the length that generated maximal twitch force. TA muscles were subjected to different force frequencies (tetani with increasing stimulation frequencies at 1–180 Hz every 500 ms with 1-min recovery intervals). All experiments were performed at 25 °C. Data acquisition and analysis were performed using LabChart Pro Software (Version 8; AD instruments, Pty Ltd.). Muscle wet weight was measured at the end of each experiment.

**Table S1. Antibodies used for immunoblot, immunofluorescence and immunohistochemistry.**

| **Product** | **Cat. number** | **Company** |
| --- | --- | --- |
| Alpha-Tubulin antibody | sc23948 | Santa Cruz |
| CRLS1 Polyclonal Antibody | PA5-100137 | Invitrogen |
| CRLS1 antibody (Internal Region) | ABIN6261040 | antibodies-online |
| Total OXPHOS Rodent WB Antibody Cocktail | ab110413 | Abcam |
| Laminin Antibodies | L9393 | Sigma |
| Laminin Monoclonal Antibody (A5) | MA1-06100 | Invitrogen |
| Recombinant anti-Myhc/MHC | AB37484 | Abcam |
| GFP Polyclonal Antibody, Alexa Fluor™ 488 | A21311 | Invitrogen |
| Goat anti-Rabbit IgG (H+L) Secondary Antibody, HRP | 31460 | Invitrogen |
| Goat anti-mouse IgG (H+L) Secondary Antibody, HRP | 31430 | Invitrogen |
| Goat anti-Rat IgG (H+L) Highly Cross-Adsorbed Secondary Antibody, Alexa Fluor™ Plus 594 | A48264 | Invitrogen |
| Goat anti-Rabbit IgG (H+L) Highly Cross-Adsorbed Secondary Antibody, Alexa Fluor™ Plus 594 | A32740 | Invitrogen |
| Goat anti-Mouse IgG (H+L) Highly Cross-Adsorbed Secondary Antibody, Alexa Fluor™ Plus 594 | A32742 | Invitrogen |
| Goat anti-Mouse IgG (H+L) Highly Cross-Adsorbed Secondary Antibody, Alexa Fluor™ Plus 488 | A32723 | Invitrogen |
| Goat anti-Rabbit IgG (H+L) Highly Cross-Adsorbed Secondary Antibody, Alexa Fluor™ Plus 488 | A32731 | Invitrogen |

**Table S2. Primer sets used for quantitative PCR analyses**

| **Application** | **Target** | **Sequence (5'-3')** |
| --- | --- | --- |
| SYBR qPCR | 36b4-F | AGATTCGGGATATGCTGTTGG |
| SYBR qPCR | 36b4-R | AAAGCCTGGAAGAAGGAGGTC |
| SYBR qPCR | Myh7-F | GAGCAGCAGGTGGATGATCT |
| SYBR qPCR | Myh7-R | GCTTGGCTCGCTCTAGGTC |
| SYBR qPCR | Myh2-F | AAGCGAAGAGTAAGGCTGTC |
| SYBR qPCR | Myh2-R | GTGATTGCTTGCAAAGGAAC |
| SYBR qPCR | Myh4-F | GTCACCAAAGGCCAGACG |
| SYBR qPCR | Myh4-R | ACATCTTCTCATACATGGACTTGG |
| SYBR qPCR | Ptpmt1-F | GCAACACCTCGAAGGAATGG |
| SYBR qPCR | Ptpmt1-R | GAGATTGGCCAAGGTTGGGA |
| SYBR qPCR | Myh1-F | TCGCTGGCTTTGAGATCTTT |
| SYBR qPCR | Myh1-R | CGAACATGTGGTGGTTGAAG |
| SYBR qPCR | Crls1-F | GCCGCCAGCTCGTATGAAAA |
| SYBR qPCR | Crls1-R | AATCAGGTAGCCCAACACGG |
| SYBR qPCR | Pnpla8-F | TTTGCCCCGGATCGATTTGT |
| SYBR qPCR | Pnpla8-R | TGTAGCCTGCAGTCCTTTGG |
| SYBR qPCR | Lclat1-F | CTGTCTCGCCCCAGTGTC |
| SYBR qPCR | Lclat1-R | TCCATGACACCATGATTCTGAC |
| SYBR qPCR | Tafazzin-F | GGGTGCACATCTTCCCAGAA |
| SYBR qPCR | Tafazzin-R | GCCGTCCAATTCCTACCCAT |
| SYBR qPCR | Cox8b-F | CTTCCGCCGTGGAGCAG |
| SYBR qPCR | Cox8b-R | GTGGGCTAAGACCCATCCTG |
| SYBR qPCR | Cox7a1-F | ATCCGGAGTCTTAGAACAGGTC |
| SYBR qPCR | Cox7a1-R | GGTCATTGTCGGCCTGGAAG |
| SYBR qPCR | Cox7a2-F | CGAAGGCATTTTGAAAACAAGGTTC |
| SYBR qPCR | Cox7a2-R | ACCAAGCGTCAGAGCCATTGTG |
| SYBR qPCR | Cox7b-F | GGTGAATTTGCACCAAGGCA |
| SYBR qPCR | Cox7b-R | TTGCTGAATGCTTCGAACTTGG |
| SYBR qPCR | Cox6c-F | CGCTGCCTATAAGTTTGGCG |
| SYBR qPCR | Cox6c-R | CCAGCCTTCCTCATCTCTTCG |

**Supplementary Figures**

**Supplementary Fig 1. Immunoblots of mitochondrial complex in young and old Soleus skeletal muscle**

**
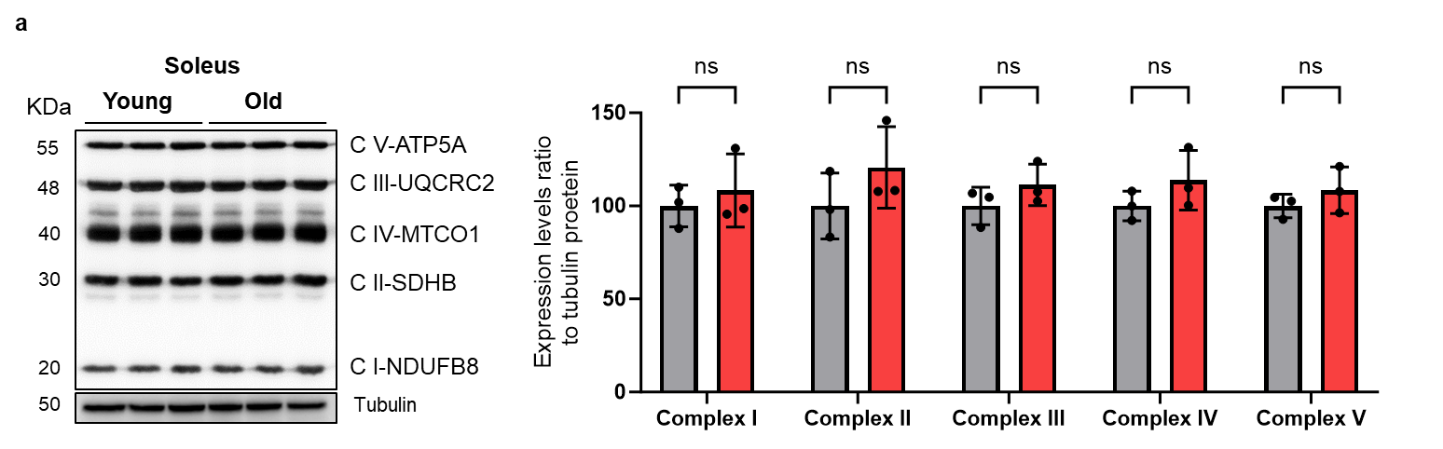
**

a Immunoblot of mitochondrial complex in young and old of soleus skeletal muscle. Ratio of mitochondrial complex protein level to tubulin in young and old of soleus skeletal muscle (n=3 per group).

Graphs presented as the mean of n ≥ 3 independent experiments with SD error bars. P values were calculated using unpaired student t-test. *P < 0.05; **P < 0.01; ***P < 0.001; n.s; no significant. Abbreviations: C57Bl/6j young (4 to 5 mo.) mice were used. C57Bl/6j aged (>22 mo.) mice were used

**Supplementary Fig 2.  Restoration of Crls1 promotes IMF mitochondrial morphology in TA muscles of young and old mice.**


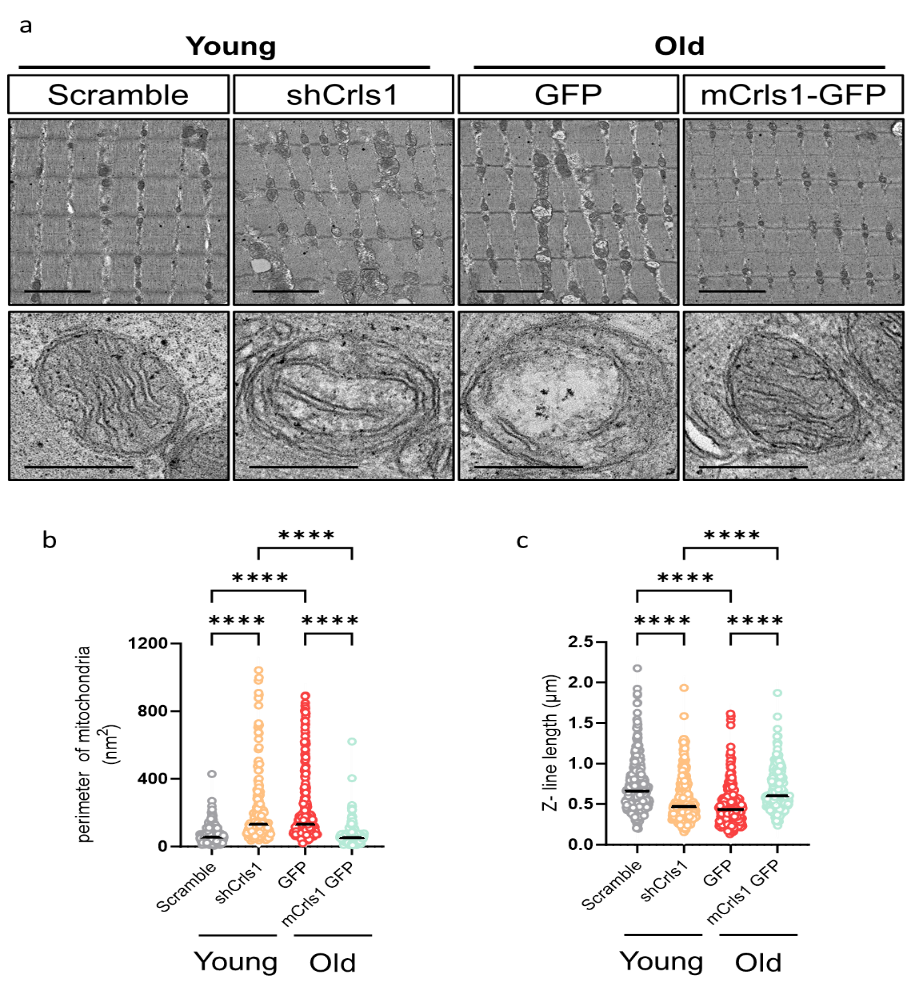


a shows representative transmission electron micrographs of interfibrillar (IMF) mitochondria in the tibialis anterior (TA) muscle of young and old mice, treated with different constructs: Scramble (control), shCrls1 (Crls1 knockdown), AAV9-GFP (green fluorescent protein control), and AAV9-mCrls1-GFP (mouse Crls1 overexpression tagged with GFP). The images display the microstructure of the muscle tissue and mitochondria. b Quantifications of IMF mitochondria perimeter. c Quantifications of Z-line size of sarcomere. Total mitochondria quantified: n = 300 scramble, n = 250 shCrls1, n = 250 aged AAV9-GFP, n = 300 aged AAV9-mCrls1-GFP. ***P < 0.001. ANOVA test with Tukey’s test. Means ± SEM. C57Bl/6 young (2 to 4 mo.) and aged (>22mo.) mice were used.

The overall implication of the figure is that the restoration of Crls1 protein in old muscles can promote healthier mitochondrial morphology, which is similar to that found in young muscles, suggesting a potential therapeutic target for age-related muscle decline.

**Supplementary Fig 3. Analyses of myogenesis gene expression in Crls1 knock down cell line**


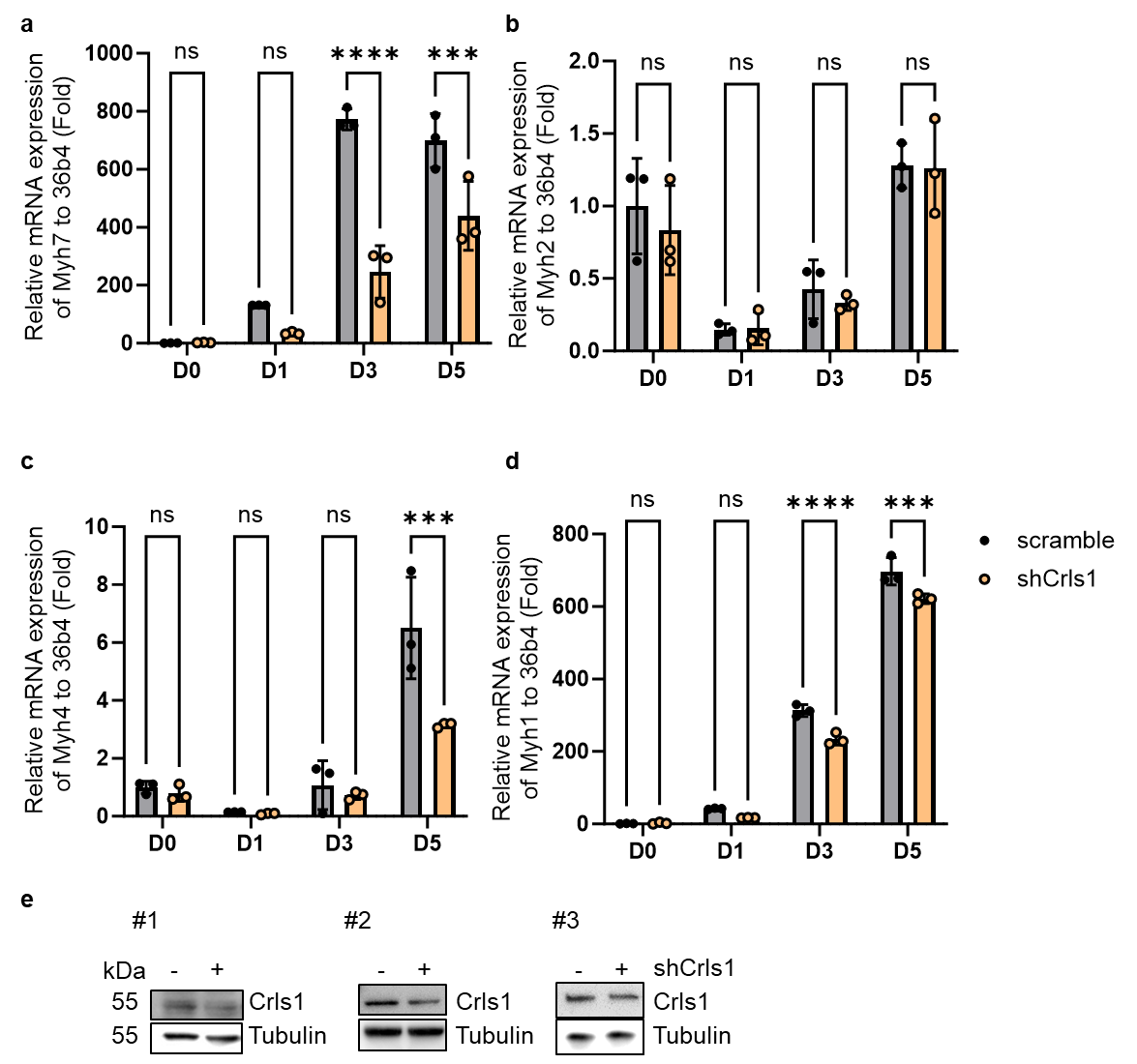


a-d qPCR analysis of myogenesis gene expression in Crls1 knock down cell line during myogenesis (n=3 per group). e Immunoblot of Crls1 in scramble and shCrls1 myoblast(n=3 per group).

-: scramble infected myoblast, +: shCrls1 infected myoblast

Graphs presented as the mean of n ≥ 3 independent experiments with SD error bars. P values were calculated using unpaired student t-test. *P < 0.05; **P < 0.01; ***P < 0.001; n.s; no significant. Abbreviations:D0; myoblast D1; differentiation for 24hours, D3; differentiation for 72hours, D5; differentiation for 120hours -: scramble infected myoblast, +: shCrls1 infected myoblast

**Supplementary Fig 4. Oxygen consumption rate and ATP production in scramble and shCrls1 infected C2C12 cell line.**


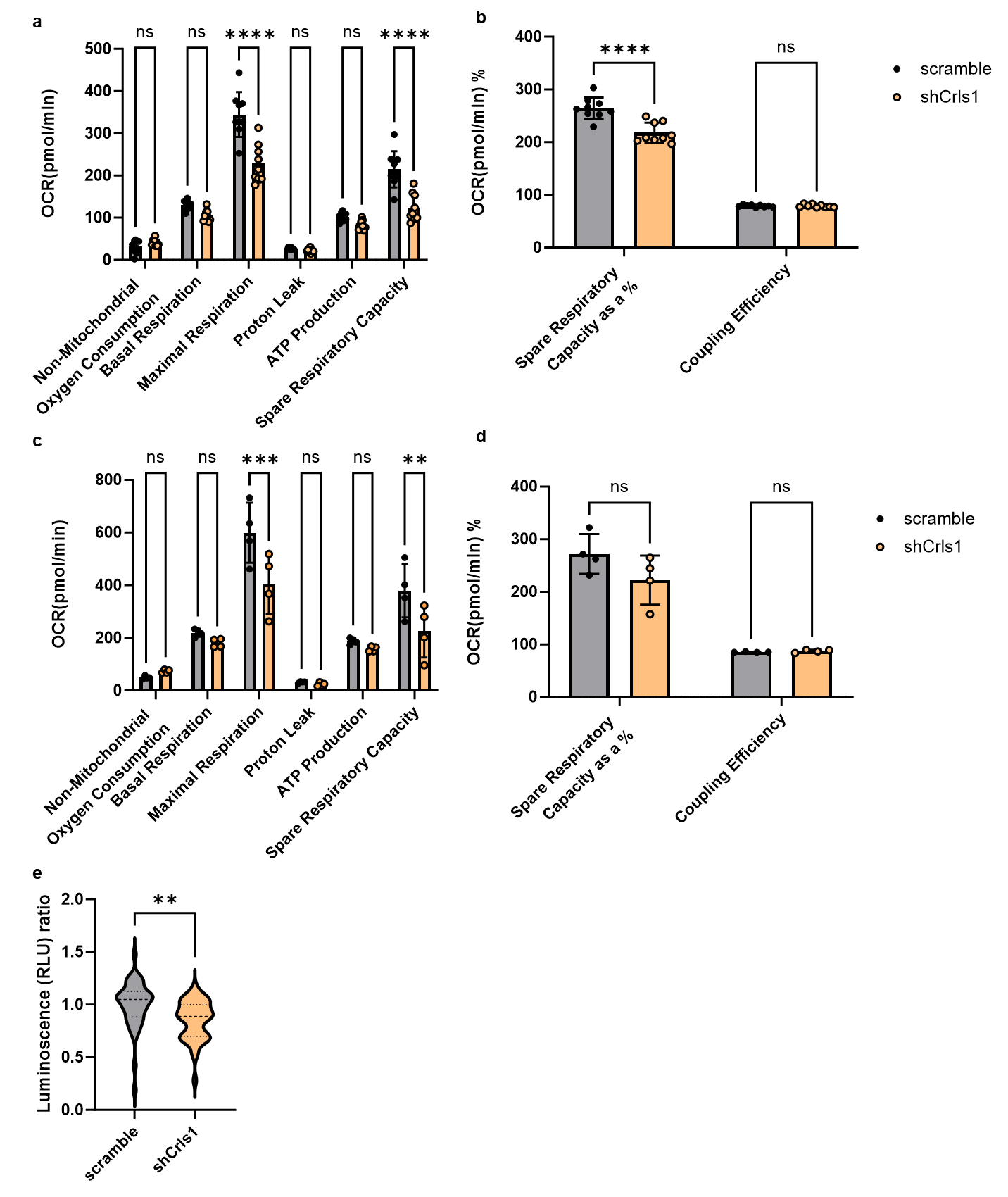


a and b. Analysis Oxygen consumption rate(OCR) in myoblast. (n=10)

c and d. Analysis Oxygen consumption rate(OCR) in differentiated myoblast for 24horus. (n=4)

e. Measure of ATP production using ATPLite Luminescence Assay system in Myoblast (n =44 per group).

**Supplementary Fig 5. Overexpression of mCrls1 in old TA muscle regeneration failure on day 7 after injury induced by cardiotoxin (CTX)**


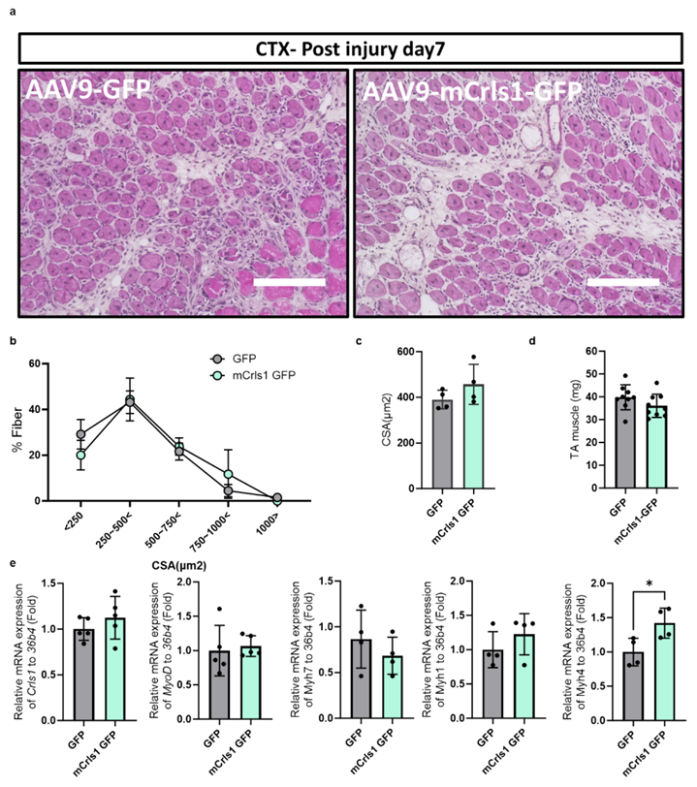


a Histological analysis, sectional tissue was stained by H&E in virus infected and regenerated for 7days TA muscle. Scale bar: 50 micrometer (AAV9-scramble and AAV9-shCrls1 group was infected by AAV9(virus diluted in PBS virus total 1x 1012 GC injected by i.m injection). b Myofiber cross-sectional areas (CSA) in GFP and mCrls1-GFP virus infected TA muscle (n=4 per group). Virus infected and regenerated old TA muscle weight (n=5). c Mean of CSA fiber in TA muscle (n=4 per group). d Virus infected and regenerated old TA muscle weight (n=7). e qPCR analysis of Crls1, MyoD, Myh7, Myh1 and Myh4 genes in virus infected young TA muscle (day7)

In the context of regeneration, although the extent of regeneration varies depending on the amount of proliferation of myoblasts, we tried to evaluate the differentiation ability of proliferated myoblasts and to observe the regenerative effects at the earlier time point than day 7 or 14. However, in the old group, tissues with AAV9-mCrls1 constructs did not show overexpression at 7 days, in which the AAV9-mCrls1 constructs seemed to be not worked yet (Sup Fig 4). That’s why we just have shown the results at 14 days.

The results suggest that the expression of the mCrls1 gene may facilitate muscle repair post-injury, as indicated by increased CSA and a significant rise in eMyHC expression in the mCrls1-GFP treated group. These findings could be valuable for strategies aiming to enhance muscle regeneration following injury.
